# Gut Microbiota and Metabolites: Orchestrating Depression Pathogenesis Through the Microbiota–Gut–Brain Axis’s Neural, Immune, and Metabolic Routes

**DOI:** 10.3390/biom16091225

**Published:** 2026-08-24

**Authors:** Zhen-Zhen Dong, Wanying Zheng, Shuojie Lv, Ling Peng, Yihan Wang, Qingjing Wang

**Affiliations:** 1 Key Laboratory of Artificial Organs and Computational Medicine of Zhejiang Province, Key Laboratory of Pollution Exposure and Health Intervention of Zhejiang Province, Shulan International Medical College, Zhejiang Shuren University, Hangzhou 310015, China; jennydong@zjsru.edu.cn (Z.-Z.D.); 202310300216@stu.zjsru.edu.cn (W.Z.); 202310300220@stu.zjsru.edu.cn (S.L.); 601282@zjsru.edu.cn (L.P.); 202211003120@stu.zjsru.edu.cn (Y.W.); 2 Key Laboratory of Artificial Organs and Computational Medicine in Zhejiang Province, Institute of Translational Medicine, Zhejiang Shuren University, Hangzhou 310015, China

**Keywords:** depression, gut microbiota, gut–brain axis, HPA axis

## Abstract

Depression (major depressive disorder, MDD) is a globally prevalent, highly disabling, and complex mental disorder whose pathogenesis has not been fully elucidated. In recent years, the role of the gut microbiota in depression via the “microbiota–gut–brain axis” (MGB axis) has attracted increasing attention. A large body of evidence indicates that the gut microbiota and its metabolites can engage in bidirectional communication with the central nervous system through three core pathways—neural, immune, and metabolic—thereby profoundly influencing the onset and progression of depression. This article reviews the specific mechanisms by which the gut microbiota affects depression through the aforementioned pathways, including regulating the balance of neurotransmitters (e.g., GABA and 5-HT), mediating neuroinflammatory responses, and adjusting the levels of metabolites such as short-chain fatty acids. This study aims to provide a theoretical basis for an in-depth understanding of the pathophysiological mechanisms of depression and the development of novel microbiota-based intervention therapeutic strategies.

## 1. Introduction

According to reports from the World Health Organization (WHO), major depressive disorder (MDD) is a common neurological disease characterized by low mood, decreased interest, and other features. In severe cases, patients may experience symptoms such as slowed thinking and reduced responsiveness, which often affect an individual’s work, life, and study, making it difficult for them to concentrate [1,2]. Approximately 3.8% of the global population suffers from depression, and women are more affected by depression than men are. Depression is the main cause of the high global self-harm rate; each year, more than 700,000 people die by suicide, and it ranks as the fourth leading cause of death among individuals aged 15–29 years [3,4].

Depressive episodes occur almost every day and last for a long time, during which patients experience depressive emotions such as sadness, irritability, and emptiness. Typical symptoms include low mood, insomnia or hypersomnia, changes in appetite, fatigue, feelings of inferiority or worthlessness, excessive self-blame, and difficulty concentrating. In severe cases, symptoms such as hallucinations and delusions may appear, and even suicidal ideation or behavior may occur [5,6,7]. Although effective treatment methods for depression already exist, more than 75% of patients in low- and middle-income countries cannot access treatment [8,9]. Moreover, despite considerable research advances, our understanding of depression is still incomplete, and existing treatment methods continue to have notable limitations [10,11].

Many types of microbiota, including the gut microbiota, oral microbiota (such as *Lactobacillus* and *bacteriophages*), and blood microbiota (such as cytokines, inflammatory response factors in serum, and microbiota related to plasma proline), can affect the occurrence of depression [5,12,13]. Among these, the gut microbiota has the most significant impact, and there is a certain degree of overlap and interaction between the oral and gut microbiota. The enteric nervous system contains many neurons; in humans, there are approximately 200 million to 600 million neurons, which is more than the number of neurons in other peripheral organs and similar to the number of neurons in the spinal cord. Research on the gut microbiota provides an emerging and highly valuable direction for our study of MDD. The gut microbiota communicates with the brain through the gut–brain axis, thereby exerting a regulatory effect on the development of depression. Recent large-scale multi-omics studies have further consolidated the link between gut microbiota composition, functional metabolic pathways, and depression phenotypes, shifting the field from descriptive association toward mechanistic dissection and subtype stratification [14,15]. In addition, depression can affect the composition of the gut microbiota.

Currently, the gut microbiota is believed to influence the occurrence and development of depression through the nervous system, immune system, and blood circulatory system. The gut microbiota is involved in processes such as digestion and absorption, immune regulation, and metabolic control. In summary, the gut microbiota can alleviate the decline in immunity, microbial imbalance in the body, and low mood caused by depression, and it can even ensure the normal daily life of the human body and the normal activities of the brain. Therefore, the gut microbiota is not only related to the occurrence and development of depression but also serves as an important link in the treatment of depression. This article reviews the bidirectional gut microbiota–depression axis and the therapeutic potential of microbiota-targeted interventions, with particular emphasis on metabolite-mediated integration of neural, immune, and metabolic pathways, and a critical distinction between preclinical causality and human correlational evidence in major depressive disorder.

## 2. The Mechanism of Depression: The Microbiota–Gut–Brain Axis (MGB)

Trillions of microorganisms in the gut, together with other substances, constitute the intestinal internal environment and coevolve with humans. A growing body of evidence indicates that the microbiota plays a crucial regulatory role in the host’s physiological functions and behaviors. Research on the gut microbiota and depression has proposed the concept of the “microbiota–gut–brain axis,” which means that the gut microbiota can participate in the regulation of nervous system function and behavior by mediating metabolic reactions, immune functions, and neural pathways [16,17,18].

## 3. The Microbiota Exacerbates the Occurrence and Development of Depression Through the Gut–Brain Axis

**Figure 1:**
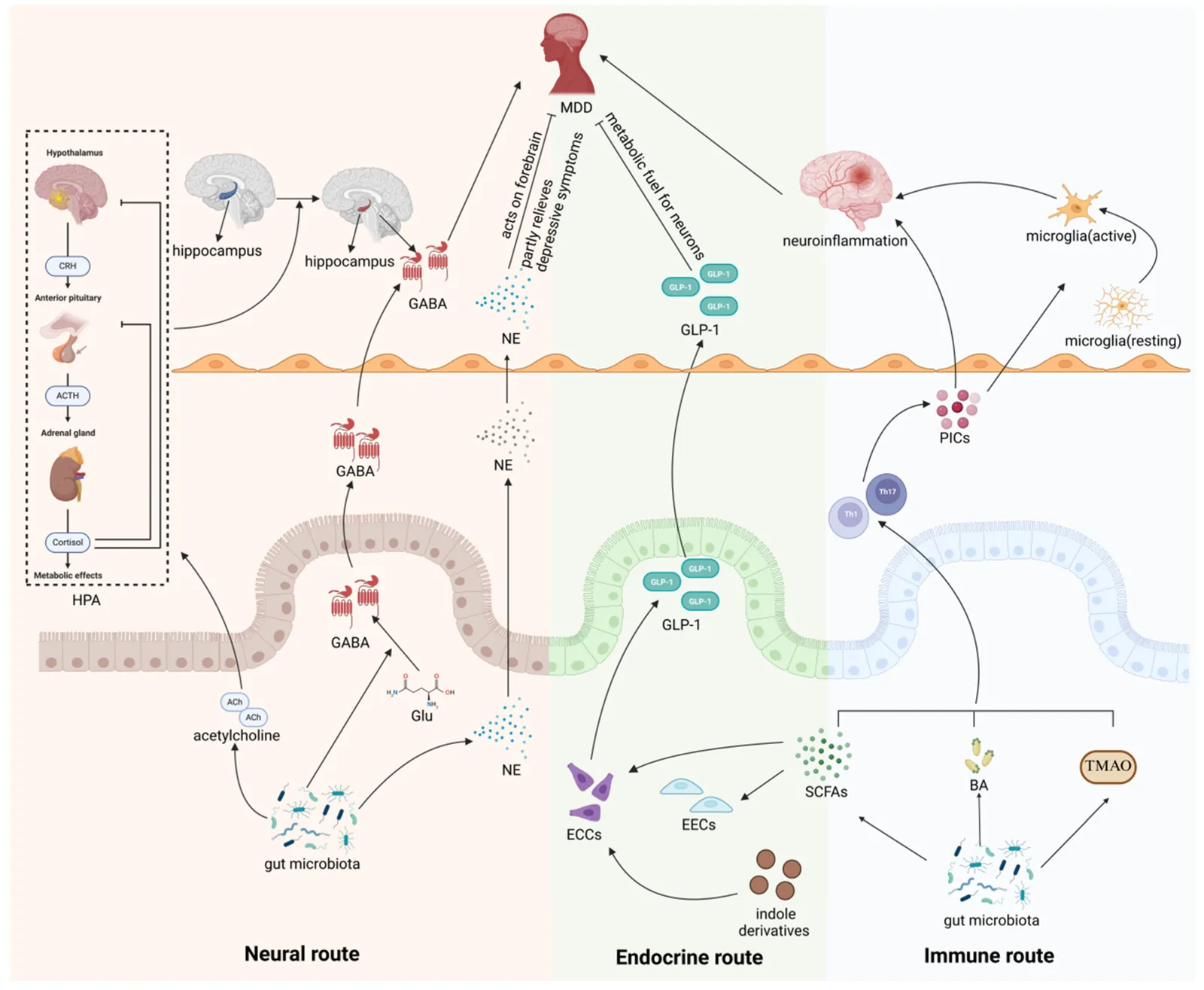
The gut microbiota influences depression via three integrated axes: immunoregulation, neural signaling, and metabolic homeostasis. Neural route (red): Microbe-derived neurotransmitters and neuromodulators—dopamine, acetylcholine (ACh), histamine, secondary bile acids, and GABA—enter the circuit. ACh stimulates the hypothalamic–pituitary–adrenal (HPA) axis; chronic negative feedback shrinks the hippocampus, weakening its GABAergic brake and thus intensifying depression. Simultaneously, bacterial GABA can reach the brain and further suppress hippocampal inhibition. Conversely, microbe-linked norepinephrine (NE) acts on select forebrain areas and may partly relieve depressive symptoms. Endocrine route (green): Indole derivatives and short-chain fatty acids (SCFAs) signal through enteroendocrine cells (EECs) and enterochromaffin cells (ECCs) to release glucagon-like peptide-1 (GLP-1) and other neuropeptides that serve as metabolic fuel for neurons, thereby influencing mood. Immune route (blue): Microbial metabolites—SCFAs, secondary bile acids and trimethylamine N-oxide (TMAO)—activate circulating immune cells, trigger proinflammatory cytokines and switch microglia from surveillant to reactive phenotypes. The resulting neuroinflammation propagates depressive changes throughout the limbic system. MDD: major depressive disorder; GLP-1: glucagon-like peptide-1; CRH: corticotropin-releasing hormone; ACTH: adrenocorticotropic hormone; PICs: post-inflammatory cytokines; NE: norepinephrine; BA: bile acid; ACh: acetylcholine; Glu: glutamate; LPS: lipopolysaccharide; SCFAs: short-chain fatty acids; EECs: enteroendocrine cells; ECCs: enterochromaffin cells; TMAO: trimethylamine N-oxide; HPA axis: hypothalamic–pituitary–adrenal axis; GABA: γ-aminobutyric acid; Th1: T helper 1 cell; Th17: T helper 17 cell. Created in BioRender. Zheng, W. (2026) https://BioRender.com/39ybfjm.

Major depressive disorder is a heterogeneous condition that, based on clinical course, can be delineated into single-episode and recurrent trajectories; however, modern research underscores that its symptomatological and etiological subtyping is not limited to this distinction alone [19,20]. It is generally believed that the gut exerts an effect on depression through ‘gut–brain interaction’ (Figure 1). The gut microbiota has a very high degree of participation and plays a significant role in the gut–brain axis. Gut–brain interaction occurs primarily through neural, endocrine, and immune pathways [21]. Under physiological conditions, the gut–brain axis is responsible for digestion and absorption, immune function, and the perception of visceral stimuli and emotional responses [22]. The gut microbiota participates in these processes through the production of neuroactive metabolites, microbial products, and signaling via neural, endocrine, and immune routes (Figure 1).

## 4. Neural Pathway: Microbiota Releases Neurotransmitters and Neuroactive Metabolites to Affect Depression

**Figure 2:**
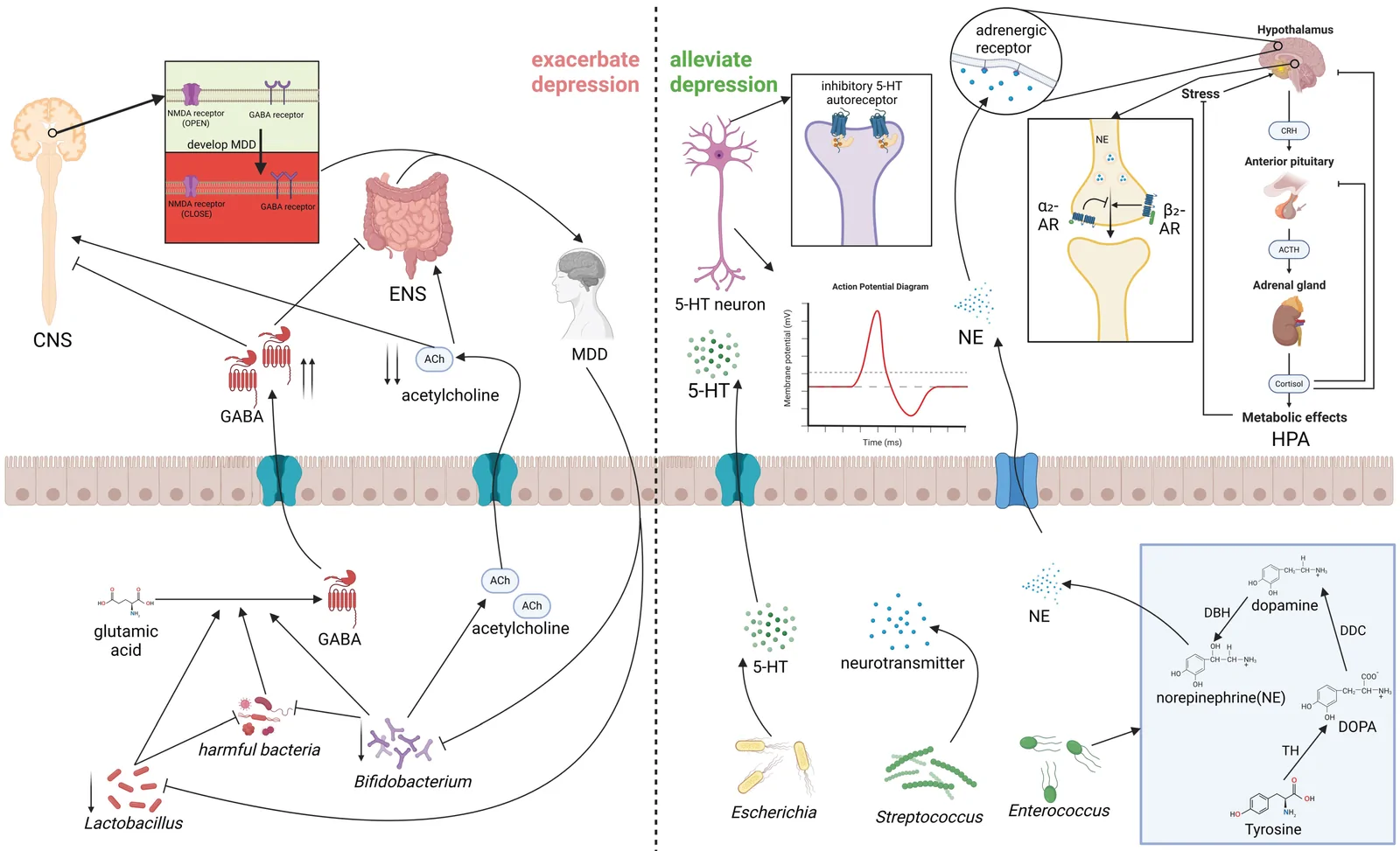
The microbiota can exacerbate or alleviate depression through neural pathways. In the red (depressive) panel, probiotic abundance declines, permitting pathobionts to expand; this shifts the GABA/ACH ratio toward higher GABA, so inhibitory neurotransmission predominates and excitability in both the CNS and ENS decreases. Consequently, GABA and NMDA receptors in specific brain regions are remodeled, increasing depressive symptoms. In the green (protective) panel, certain commensals synthesize and release NE within the gut; NE crosses the blood–brain barrier, targets discrete brain areas, and acts via the HPA axis to attenuate depression. Simultaneously, microbes secrete 5-HT, which engages 5-HT receptors on raphe neurons, modulates their firing, and sustains serotonergic homeostasis and the autoinhibitory tone, collectively alleviating depression. MDD: major depressive disorder; HPA axis: hypothalamic–pituitary–adrenal axis; 5-TH: 5-hydroxytryptamine; GABA: gamma-aminobutyric acid; NE: norepinephrine; CNS: central nervous system; ENS: enteric nervous system; α_2_-AR: α2-adrenergic receptor; β_2_-AR: beta-2 adrenergic receptor; TH: tyrosine hydroxylase; DDC: aromatic L-amino acid decarboxylase; DBH: dopamine β-hydroxylase; NMDA:N-methyl-D-aspartate; ACH: acetylcholine; DOPA: dihydroxyphenylalanine; ACTH: adrenocorticotropic hormone; CRH: corticotropin-releasing hormone. Created in BioRender. Zheng, W. (2026) https://BioRender.com/y8fccws.

The microbiota interacts directly with these afferent neurons by releasing neurotransmitters in the gut. In vitro cultivation studies and animal models have demonstrated that these neurotransmitters can stimulate intrinsic primary afferent neurons in the gut or directly stimulate extrinsic primary afferent neurons in the vagus nerve, pelvic nerves, and spinal nerves: *Bifidobacterium* and *Lactobacillus* can produce acetylcholine and γ-aminobutyric acid (GABA), whereas *Escherichia*, *Streptococcus*, and *Enterococcus* can produce serotonin (5-hydroxytryptamine, 5-HT), dopamine, and norepinephrine (NE). Conversely, microbial populations that express GABA, NE, and 5-HT receptors can receive neurotransmitters released by the brain through motor signals or efferent signals [23,24,25,26,27] (Figure 2) (Table 1).

The bidirectional connections between the gut and the brain include the vagus nerve, the hypothalamic–pituitary–adrenal (HPA) axis, and other components. The HPA axis represents a critical neuroendocrine interface between the gut microbiota and depression [36,37]. In major depressive disorder, chronic stress-induced HPA axis hyperactivity is frequently coupled with glucocorticoid resistance, characterized by reduced glucocorticoid receptor (GR) sensitivity and impaired negative feedback, resulting in sustained cortisol elevation and exacerbation of neuroinflammation [38]. The gut microbiota modulates this axis through several mechanisms: microbial metabolites such as short-chain fatty acids (SCFAs) may influence GR expression and HPA axis responsiveness in limbic and cortical regions, while microbiota-derived lipopolysaccharides (LPS) and proinflammatory cytokines can further compromise GR-mediated feedback inhibition by sustaining neuroinflammatory signaling [39,40]. Moreover, specific commensals (e.g., *Lactobacillus* and *Bifidobacterium*) have been reported to attenuate HPA axis hyperreactivity in animal models, though human trials have yielded mixed results regarding cortisol modulation [41]. Nevertheless, direct causal evidence linking specific microbial configurations to glucocorticoid resistance in human MDD is still insufficient and requires further validation [22,42].

The regulation of the gut–brain axis relies mainly on the vagus nerve present in the human digestive tract, which transmits signals from the gut to the central nervous system for integration, thereby generating adaptive changes. Among these changes, maladaptive responses are mainly manifested as gastrointestinal diseases and neurodegenerative diseases. In addition, the enteric nervous system (ENS) and the extrinsic nervous system or central nervous system (CNS) can jointly regulate intestinal activities and the microbiota.

Certain specific microorganisms can synthesize and/or regulate a variety of neurotransmitters involved in nerve conduction [43], and they may also produce metabolites (such as short-chain fatty acids) that directly or indirectly affect neural activity [44]. Some pathogenic bacteria and commensal bacteria use the local action of the enteric nervous system (ENS) to create a more suitable environment for their survival or promote the production of their toxins [45], resulting in maladaptive symptoms and causing the ENS to produce inflammatory factors. These inflammatory factors are transmitted to the dorsal motor nucleus of the vagus nerve in the brainstem medulla through the vagus nerve and the nucleus tractus solitarius, thereby affecting the central nervous system (CNS) [46]. In this way, a mutually influential neural pathway is formed, which affects the occurrence of depression.

### 4.1. Imbalanced Ratio of Acetylcholine to γ-Aminobutyric Acid (GABA) Exacerbates Depression

Neurotransmitters are effective in small amounts. Moreover, depression can significantly change the composition of the gut microbiota. For example, in patients with depression, the abundance of probiotics such as *Bifidobacterium* and *Lactobacillus* in the intestinal flora decreases, and the metabolites of these two types of bacteria can inhibit the growth of many harmful bacteria. When depression occurs, the abundance of probiotics decreases, leading to harmful bacteria occupying a dominant position. Both the host and bacteria have the ability to convert glutamic acid into GABA, which leads to the massive release of GABA, while the release of acetylcholine does not increase but instead decreases.

Acetylcholine is an excitatory neurotransmitter; its reduced content allows inhibitory neurotransmitters to occupy a dominant position, which lowers the excitability of the central nervous system (CNS) and the enteric nervous system (ENS). This causes the gut to remain in an inhibited state for a long time, and under the long-term influence of this state, the central nervous system also exhibits a depressed state. Changes in the gut microbiota lead to changes in γ-aminobutyric acid receptors and NMDA receptors in specific brain regions, thereby exacerbating depression.

GABA is an amino acid neurotransmitter and an inhibitory neurotransmitter in the central nervous system (CNS). It is produced mainly by model organisms such as *Escherichia coli*; pathogenic bacteria such as *Listeria monocytogenes*; and several genera, including *Bifidobacterium* and *Lactobacillus* (e.g., *Lactobacillus*, *Lactococcus lactis*, and *Streptococcus thermophilus*) [47]. Compared with healthy controls, patients with depression have lower cortical GABA concentrations and neuronal density, and enzyme synthesis in the peripheral system, and cerebrospinal fluid (CSF) decreases, which reduces the excitability of the nervous system to GABA [45,48]. Therefore, GABA is also used as a targeted drug for the treatment of depression [49,50,51,52].

### 4.2. Catechols, 5-Hydroxytryptamine (5-HT), and Norepinephrine (NE) Produced by Microbiota Alleviate the Occurrence of Depression

Both 5-hydroxytryptamine (5-HT) and norepinephrine (NE) are monoamine neurotransmitters. The synthesis of norepinephrine starts with dopamine: dopamine (DA) is derived from tyrosine. First, tyrosine is converted into DOPA under the action of tyrosine hydroxylase (TH), then DOPA is converted into dopamine under the action of DOPA decarboxylase (DDC), and finally, dopamine is converted into norepinephrine under the action of dopamine β-hydroxylase (DBH) [53]. NE exerts its effects through adrenergic receptors in the central nervous system. Through the HPA axis, α2-adrenergic receptors located on the presynaptic membrane of nerve terminals can inhibit the release of NE, whereas presynaptic β2-adrenergic receptors promote the release of NE when activated.

The activation of inhibitory 5-HT autoreceptors regulates the firing activity of 5-HTergic neurons, maintaining the homeostasis of the serotonergic system and the self-inhibitory mechanism of neuronal activity. Increasing the transmission intensity of both 5-HT and NE can reduce the risk of depression [45,53,54].

Catecholamines play various roles in host physiological processes, including the stress response and gut-related functions, thereby affecting host behavior and decision-making. The catecholamine system is the first system proven to mediate host–microbe interactions and has important clinical significance. For example, enteric nerves can synthesize dopamine and norepinephrine but lack phenylethanolamine-N-methyltransferase, the enzyme that converts norepinephrine into epinephrine. Among them, the microbiota has been proven to play a key role in converting the inactive forms of host-derived norepinephrine and dopamine into biologically active forms. Multiple experiments have confirmed that *Escherichia coli*, *Streptococcus*, and *Enterococcus* play important roles in the production of dopamine and norepinephrine [55].

It should be noted that existing evidence linking the “gut microbiota–neural pathway–depression” axis remains predominantly derived from in vitro experiments and animal studies, with direct causal evidence at the human level still insufficient. Moreover, the impact of microbiota-derived neurotransmitters on the central nervous system is more likely mediated through indirect pathways involving the enteric nervous system, vagus nerve, immune signaling, and endocrine mechanisms, rather than through a simplistic direct-action model.

## 5. Immune Pathway: Microorganisms Contribute to the Pathophysiology of Depression by Modulating Inflammatory Responses

**Figure 3:**
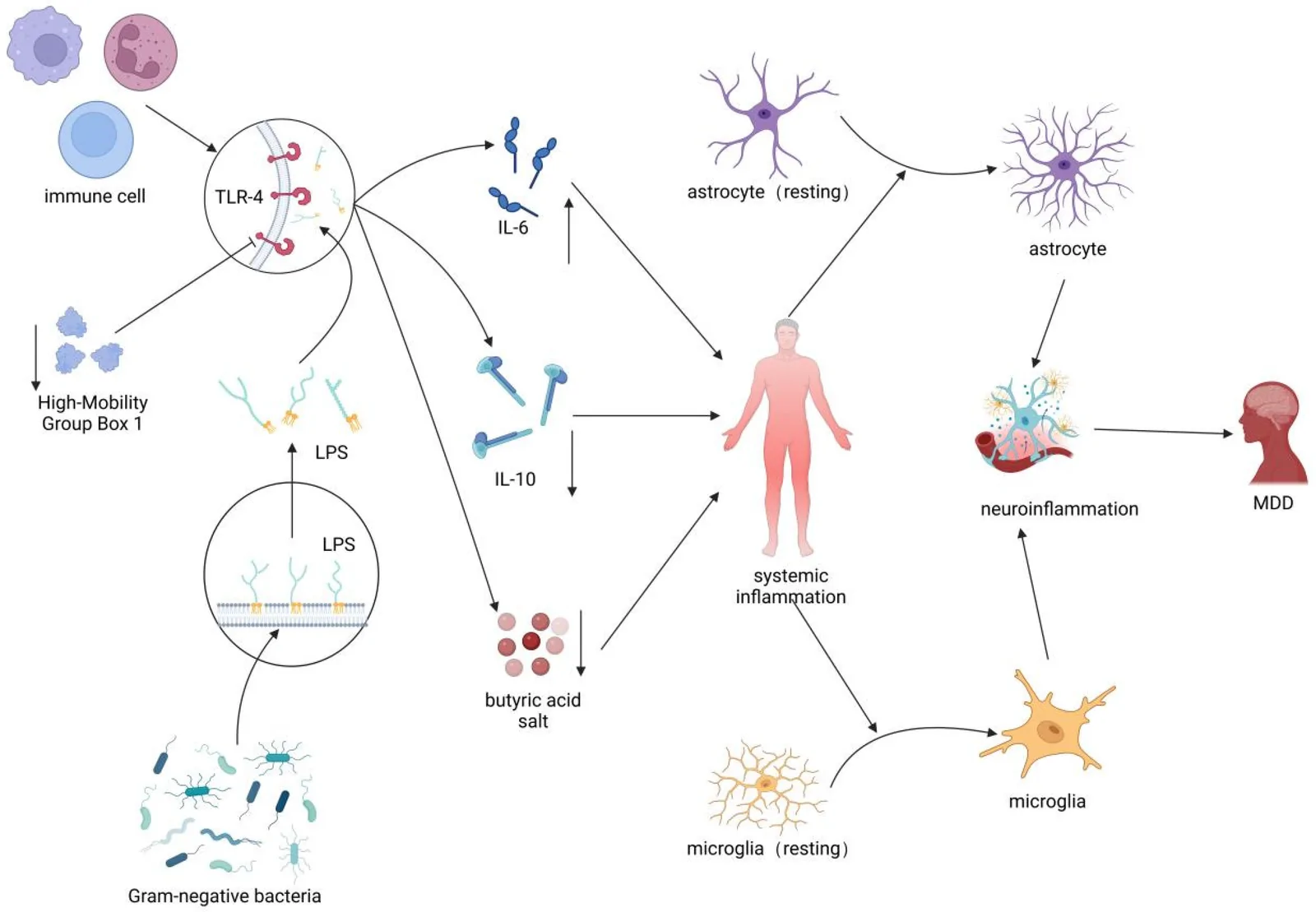
Microorganisms modulate depression via immune pathways. In patients with major depressive disorder (MDD), HMGB1 in the bloodstream upregulates TLR-4 on immune cells. When intestinal barrier dysfunction permits bacterial translocation, LPS from the outer membrane of Gram-negative bacteria enters the circulation. While passing through lymphoid tissue, this LPS binds to TLR-4, triggering a rapid surge of proinflammatory cytokines (e.g., IL-6) and a parallel decrease in the levels of anti-inflammatory mediators (e.g., IL-10 and butyrate). The resulting systemic inflammation activates resting microglia and astrocytes, converting them to a reactive phenotype that releases proinflammatory signals within the brain. This neuroinflammation ultimately amplifies depressive symptoms. HMGB1: high-mobility group box 1; MDD: major depressive disorder; TLR-4: Toll-like receptor 4; IL-6: interleukin-6; IL-10: interleukin-10; LPS: lipopolysaccharide. Created in BioRender. Zheng, W. (2026) https://BioRender.com/ko8fofp.

The human gut hosts nearly 3000 species of microorganisms, among which bacteria occupy a dominant position—approximately 90% of the gut microbiota are bacteria. Bacteria can produce quorum-sensing (QS) molecules, which play a key role in establishing the structure of the gut microbiome at the species and strain levels [56,57]. They are also used for communication with the host and affect human physical and mental health. The microbial signaling molecules produced by bacteria can activate the receptors of intestinal cells, trigger the sensing of gene expression, and thereby influence the occurrence and development of neurological diseases such as depression [56] (Figure 3) (Table 2).

The gut microbiota has a significant effect on the activity and function of the immune system and possesses immune regulatory functions [68,69]. It regulates the level of cytokines through interactions with the lymphoid tissue of the digestive tract, triggering systemic inflammation and increasing the level of cytokines in the blood. This finding also indicates that depression is a systemic disease [69,70]. Systemic inflammation can also trigger neuroinflammation and activate microglia and astrocytes, thereby promoting the development of major depressive disorder (MDD) and affecting behavior and mood.

The feces of patients with major depressive disorder (MDD) present relatively high abundances of *Bilophila* (Pseudomonadota) and *Bacteroides* (Bacteroidota) but relatively low abundances of *Anaerobutyricum* and *Dialister* (Bacillota); however, these microbial alterations are not fully consistent across independent studies [71,72,73]. This heterogeneity is primarily attributable to variations in sequencing and analytical methodologies, differences in case definitions and sample characteristics, and insufficient control for confounders such as diet and antidepressant use. Both *Bilophila* and *Bacteroides* are Gram-negative bacteria; therefore, when intestinal dysfunction leads to bacterial translocation, the lipopolysaccharides (LPS) on their cell membranes can stimulate the innate immune system by activating TLR-4 (Toll-like receptor 4). *Anaerobutyricum* is a genus of Gram-positive anaerobic bacteria belonging to Bacillota [74]. Bacteria of the genus *Anaerobutyricum* can metabolize carbohydrates to produce butyrate, a short-chain fatty acid that is crucial for intestinal homeostasis and has immunosuppressive and anti-inflammatory functions [75].

In addition, high-mobility group box 1 (HMGB1), an important mediator of innate immunity, plays a role as an “alarm” or “danger” signal in LPS-induced depressive-like behavior, and the plasma level of HMGB1 in patients with MDD is relatively low. The decrease in HMGB1 levels can explain the high expression of TLR-4 detected in patients with recurrent major depressive disorder (r-MDD): as the plasma level of the ligand (HMGB1) decreases, the expression level of the receptor (TLR-4) increases through a compensatory mechanism. Elevated LPS levels may also lead to increased TLR-4 expression: patients with r-MDD were previously diagnosed with acute major depressive disorder (a-MDD), so increased LPS levels during a-MDD may activate TLR-4, resulting in increased TLR-4 expression during the r-MDD phase [71,76].

*Bacteroides* can also activate T-cell-mediated responses through interactions with the immune system, thereby inhibiting specific inflammatory responses. By producing zwitterionic polysaccharides, *Bacteroides* activate CD4+ T cells, and the production of CD4+ T cells stimulates the release of interleukin-10 (IL-10), thereby inhibiting specific inflammatory responses and abscess formation. Intestinal immune protection depends on the development of *Bacteroides*, the interleukin-36 signaling pathway, and macrophages. These factors jointly maintain the overall health of the host, help alleviate neuroinflammation, and slow the occurrence and development of depression.

Gut microbiota dysbiosis is closely associated with peripheral and neuroinflammation in depression, and may participate in the pathophysiology of certain depression subtypes by modulating intestinal barrier permeability, innate immune receptor signaling, cytokine release, and glial cell activation. However, its specific causal role in human MDD, the defining microbial signatures, and the reproducible molecular mechanisms remain to be further elucidated through longitudinal cohort studies, stratified clinical investigations, and interventional trials [77,78].

## 6. Metabolic Pathway: Metabolites Produced by Microorganisms Affect the Progression of Depression (Hormones, Metabolites, or Neurotransmitters)

**Figure 4:**
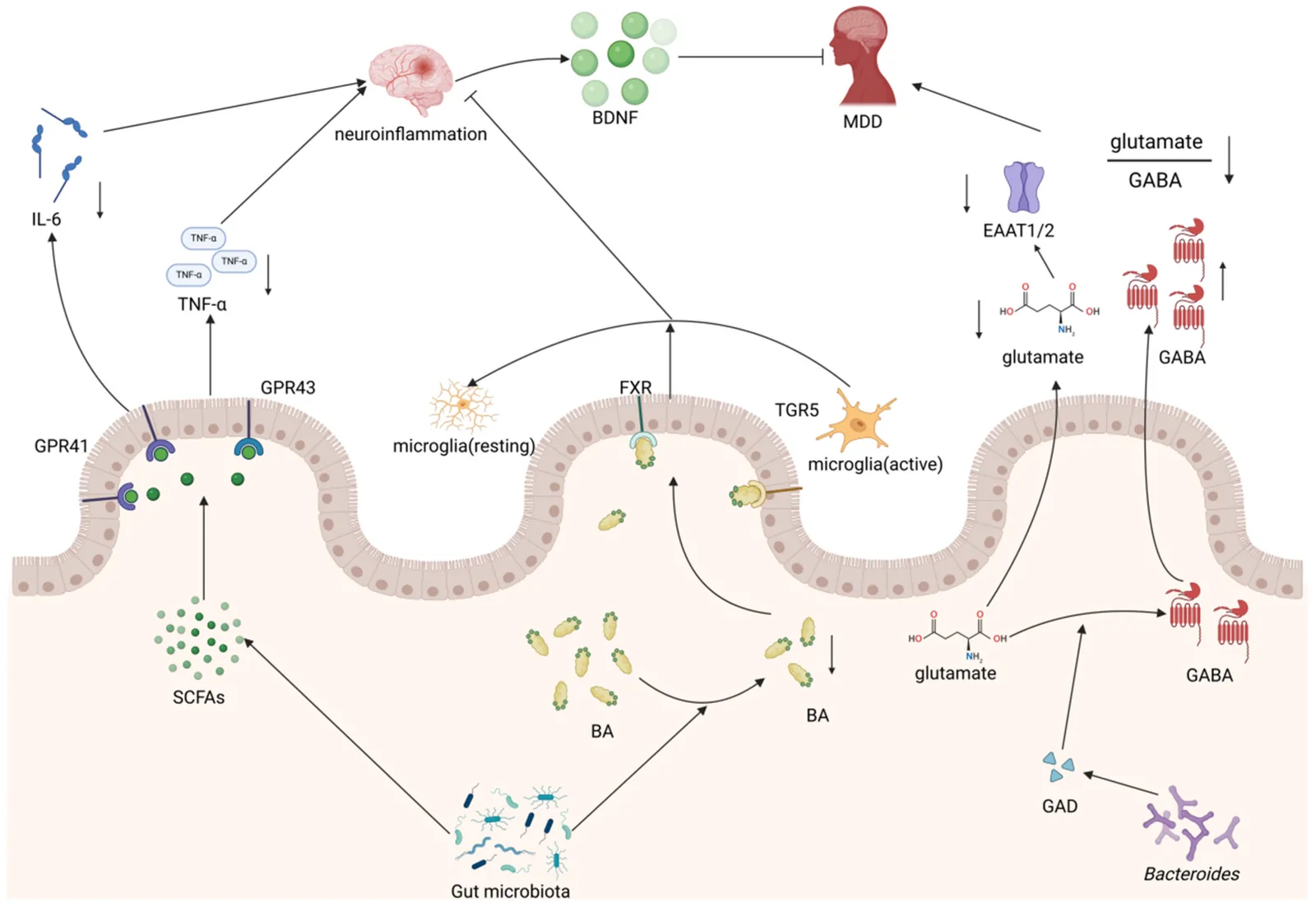
The gut microbiota influences depression through metabolic pathways. Short-chain fatty acids (butyrate, propionate, and acetate) are produced by microbial fermentation signals through GPR41/43 to entrain circadian rhythms, blood pressure, neuroimmune crosstalk, and emotional valence; their fecal concentrations are markedly lower in first-episode and treatment-resistant MDD patients and correlate inversely with symptom severity. SCFA depletion simultaneously unleashes proinflammatory cytokines that drive neuroinflammation, whereas propionate further modulates mood by stimulating dopamine synthesis. The levels of secondary bile acids, which act on FXR and TGR5, likewise decline in MDD patients and mirror their clinical severity. *Bacteroides* spp. convert glutamate → glutamine → GABA, preserving the excitatory/inhibitory balance that governs BDNF release from neurons and glia. A decrease in BDNF, coupled with glutamate excitotoxicity, triggers neuroinflammation and gliosis, ultimately reshaping neuronal circuits and deepening depressive behavior. MDD: major depressive disorder; BDNF: brain-derived neurotrophic factor; IL-6: interleukin-6; TNF-α: tumor necrosis factor-alpha; GABA: gamma-aminobutyric acid; SCFAs: short-chain fatty acids; BA: bile acids; FXR: farnesoid X receptor; TGR5: Takeda G protein-coupled receptor 5; GPCR: G protein-coupled receptor; GPR41/43: G protein-coupled receptor 41/43; EAAT1/2: excitatory amino acid transporter 1/2; GAD: glutamate decarboxylase. Created in BioRender. Zheng, W. (2026) https://BioRender.com/u7ohe07.

Short-chain fatty acids (SCFAs), including butyrate, propionate, and acetate, are key metabolites produced by gut microbial activity. They can play a central role in influencing mood states and cognitive processes through G protein-coupled receptors, with specific functions, including regulating gastrointestinal function, blood pressure, circadian rhythm, and neuroimmunity. These findings indicate that short-chain fatty acids (SCFAs) are expected to become key participants in the communication of the microbiota–gut–brain axis [44,79,80,81,82,83] (Figure 4).

The level of short-chain fatty acids (SCFAs) in the feces of depressed women is significantly lower than that in the feces of nondepressed women, which is also considered one of the potential triggers of major depressive disorder (MDD). The content of SCFAs in feces may be negatively correlated with the severity of depression. It should be noted that fecal SCFA concentrations primarily reflect intraluminal production and excretion, and their relationship with circulating serum or cerebrospinal fluid (CSF) levels is not straightforward. Systemic availability of SCFAs is influenced by intestinal absorption efficiency, hepatic first-pass metabolism, and blood–brain barrier permeability [82,84,85]. In addition, among short-chain fatty acids (SCFAs), the production of propionate is related to the monoamine neurotransmitter dopamine, and the level of dopamine is directly related to the severity of major depressive disorder (MDD) [44,86].

The role of acetate in immunity and the antidepressant effects of propionate and butyrate require more in-depth research. Patients with major depressive disorder (MDD) have bile acid (BA) metabolism disorders, and the level of bile acid (BA) is negatively correlated with the severity of depressive symptoms. Bile acid (BA) regulates the physiological and metabolic responses of an organism by binding to its specific receptors—farnesoid X receptor (FXR) and Takeda G protein-coupled receptor 5 (TGR5) [12,87,88].

Glutamatergic transmission is related to treatment-resistant depression (TRD) [89,90,91]. Patients with depression have a variety of abnormalities in the glutamatergic system, including reduced glial cell density, decreased expression of the glutamate reuptake transporters EAAT1 and EAAT2, and weakened enzymatic conversion of glutamate to glutamine. In addition, the function of the central nervous system fundamentally depends on the delicate physiological balance between the glutamatergic system and the GABAergic system.

Since the release rate of extracellular glutamate exceeds the clearance rate of glial cells, the resulting inflammation and neurodegenerative changes may lead to acute volume reduction and other cellular structural abnormalities detected in the brains of depressed patients. This impairs the glutamate reuptake function of glial cells and further promotes the conversion of glutamate to glutamine to limit excitotoxicity and provide necessary precursor substances for GABA synthesis [48,92,93,94].

*Bacteroides* can produce a large amount of GABA. Transcriptome analysis of feces from healthy individuals revealed that *Bacteroides*, *Parabacteroides*, and *Escherichia* actively express genes related to the GABA synthesis pathway. By combining 16S rRNA sequencing with functional magnetic resonance imaging (fMRI) results from patients with major depressive disorder (MDD), the relative abundance of *Bacteroides* in feces was found to be negatively correlated with brain region characteristics related to depression [95,96,97].

Neural, immune, and metabolic pathways do not operate in isolation; rather, they constitute an interconnected network in which dysregulation in one pathway may amplify dysfunction in others. Existing evidence suggests that gut microbiota-derived short-chain fatty acids (SCFAs) participate not only in host metabolism but also modulate mucosal and systemic immune responses through mechanisms including G protein-coupled receptor signaling, histone deacetylase inhibition, and immune cell function regulation, thereby contributing to central immune homeostasis to a certain extent [98,99,100]. Concurrently, lipopolysaccharide (LPS) and the proinflammatory cytokine responses it induces can promote neuroinflammation via peripheral–central immune communication and are associated with blood–brain barrier impairment, hypothalamic–pituitary–adrenal (HPA) axis activation, and neuronal dysfunction [101,102,103]. At the neurotransmitter level, current evidence primarily supports that microbial metabolites such as butyrate can modulate serotonin (5-HT)-related signaling and influence GABAergic pathways in certain animal models; however, these findings are insufficient to directly infer that such metabolites necessarily alter the synthesis of specific neurotransmitters in patients with major depressive disorder [104,105]. Therefore, the pathogenesis of major depressive disorder likely arises from the convergence of these interacting pathways rather than from any single mechanism alone [106,107].

Collectively, evidence from the microbiota–gut–brain axis provides an important perspective for elucidating the pathophysiological mechanisms of depression; however, the current evidence is more appropriately regarded as a component within a multifactorial pathological network rather than a single determinant. Its practical value in diagnostic stratification and as an intervention target for depression remains to be further validated through studies employing larger sample sizes, standardized detection protocols, and cross-species verification [12,108].

## 7. Challenges and Future Perspectives

As a major disease with global impact, depression affects a substantial population and carries a high risk of mortality. Its pathogenesis may involve multiple factors, including genetics, neuroinflammation, major infectious diseases, and other serious conditions, with many unknown areas remaining to be explored. Although research on mental disorders such as depression has advanced considerably in recent years, the specific and direct etiology of depression has yet to be fully clarified. No definitive pathogenic substances have been identified; the causal relationship between pathogenic and beneficial bacteria remains unclear; the mechanisms of the gut–brain axis have not been comprehensively elucidated; and the parallel relationship between characteristic microbiota and depressive symptoms awaits establishment.

### 7.1. Research Limitations

Existing studies are substantially constrained by methodological limitations. The majority of mechanistic evidence derives from germ-free animals, chronic stress models, fecal microbiota transplantation, and probiotic interventions, which support preclinical causality in inducing depressive-like behaviors but cannot be equated with the pathological mechanisms of human MDD [35,72,109]. In particular, germ-free animals show profound developmental and immunometabolic alterations, and rodent stress paradigms model only selected behavioral dimensions rather than the heterogeneous, symptom-defined clinical syndrome of MDD in humans [110,111,112]. Accordingly, microbiota-driven depressive-like behaviors in animals should be interpreted as mechanistically informative but not as direct equivalents of human MDD [36]. Human studies are predominantly cross-sectional observations, primarily reporting correlations among microbiota composition, SCFAs, inflammatory factors, and symptom severity, making it difficult to disentangle causal directionality [35,113]. Furthermore, research findings exhibit marked inconsistencies and heterogeneity [114]. On one hand, certain microbiota alterations in MDD are not stable and are difficult to regard as universal biomarkers. On the other hand, the same bacterial genus may possess dual proinflammatory and immunoprotective properties. For example, *Bacteroides* has been associated with inflammatory depression in clinical cohorts, yet broader immunological evidence indicates that microbiota metabolites can regulate T cells, macrophages, and mucosal immunity [115,116,117]. Consequently, a single bacterial genus cannot be simplistically categorized as either “harmful” or “beneficial” [118,119]. This discrepancy likely depends on species-specific metabolic profiles, strain-level specificity, metabolic context (e.g., GABA vs. LPS production), and host inflammatory state [120]. Specifically, different *Bacteroides* species produce distinct bioactive metabolites and exert opposite effects on depressive behavior: *B. uniformis* reduces butyric acid and promotes Th17-related inflammation, thereby increasing depression susceptibility, whereas *B. thetaiotaomicron* and *B. vulgatus* elevate short-chain fatty acids and suppress Th1/Th17 responses, attenuating depressive-like behaviors [115,120]. Even within a single species, strain-level variation is critical—*B. uniformis* CECT 7771 alleviates depressive-like behavior while *B. uniformis* ATCC 8492 induces it [115,121]. Furthermore, *Bacteroides* species synthesize a diverse array of neuroactive metabolites—including GABA, polysaccharide A, and tryptophan-derived compounds—that differently modulate immune cells and neural function [118,122,123].

### 7.2. Clinical Challenges

Although an increasing body of research has implicated gut microbiota dysbiosis, metabolic abnormalities, and immune inflammation in the pathogenesis and progression of MDD, reproducible and generalizable biomarker systems remain lacking, and stable composite indicators for objective diagnosis or subtyping of MDD have yet to be established. This limitation is closely associated with the considerable heterogeneity of existing findings, evident methodological discrepancies, and insufficient control for confounding factors [114]. Meanwhile, although fecal microbiota transplantation, probiotics, and dietary interventions have shown preliminary promise in animal experiments and early-stage clinical studies, the overall therapeutic efficacy in human studies remains modest at present, with substantial variations in intervention protocols, target populations, outcome measures, and follow-up durations [12]. Consequently, their clinical application continues to depend upon more rigorous placebo-controlled trials and mechanistic validation [12,124]. Furthermore, MDD itself exhibits significant clinical and biological heterogeneity; different subtypes or comorbid conditions—including inflammatory depression, recurrent depression, MDD comorbid with irritable bowel syndrome, MDD comorbid with overweight or obesity, and bipolar depression—may differ in microbiota composition, metabolic pathways, and immune profiles [14]. This suggests that unified microbiota-targeted intervention strategies are unlikely to be feasible in the future, and that precision research and individualized treatment approaches based on subtype stratification will be required instead [125].

### 7.3. Future Directions

Future research should shift from cross-sectional association analyses to longitudinal cohort studies and mechanistic clinical investigations, integrating microbial composition, metabolite profiles, inflammatory markers, intestinal barrier function, and neuroimaging evidence in a coordinated manner [14,126]. Concurrently, the research focus should move beyond simple comparisons of bacterial abundance toward functional module and key pathway analyses, with particular emphasis on short-chain fatty acids, the tryptophan–kynurenine pathway, GABA, bile acids, and lipid and energy metabolism-related pathways, as existing evidence suggests that depression-associated abnormalities are more accurately characterized as functional imbalances rather than alterations in single bacterial genera [14,127]. At the clinical translation level, a more realistic direction is to develop adjunctive interventions based on depression subtype stratification, rather than replacing existing antidepressant therapies in the short term [12,127]. Although fecal microbiota transplantation, probiotics, and dietary interventions have shown certain potential, their therapeutic durability, standardized protocols, and applicable populations require further validation [124,128]. Moreover, comorbid conditions such as irritable bowel syndrome, obesity, or bipolar depression may correspond to distinct microbial and metabolic profiles [126,129]. Among the proposed subtypes, inflammatory depression and depression comorbid with irritable bowel syndrome (IBS) represent particularly promising initial targets for mechanistic and interventional studies, given the comparatively robust evidence linking peripheral immune activation and intestinal barrier dysfunction to microbiota alterations in these populations [77,130]. With respect to metabolic pathways, the tryptophan–kynurenine axis warrants priority validation, as it simultaneously intersects immune regulation, neurotransmitter synthesis, and HPA axis activity [131,132]. Short-chain fatty acid (SCFA) signaling also warrants priority validation for its roles in immune regulation and neurotransmitter synthesis, though direct evidence for SCFA–HPA axis coupling remains primarily preclinical [133,134]. Regarding personalized intervention strategies, future clinical trials should consider stratifying participants based on baseline inflammatory profiles (e.g., C-reactive protein, IL-6) or gut permeability markers to identify subpopulations most likely to respond to microbiota-targeted therapies [77,135]. In summary, the critical challenge is not to repeatedly demonstrate that the microbiota is “associated” with depression, but to identify which immune and metabolic pathways exhibit stable reproducibility, causality, and clinical translational value in specific depression subtypes.

## 8. Conclusions

Major depressive disorder (MDD) is a globally prevalent neurological disease with a high mortality rate, and its pathogenesis involves multiple factors including genetics, neuroinflammation, and major infectious diseases. The gut microbiota plays a pivotal role in the onset and progression of depression through the microbiota–gut–brain axis, primarily via neural, endocrine, immune, and metabolic pathways. Specifically, alterations in the gut microbiota composition affect the synthesis and balance of neurotransmitters such as γ-aminobutyric acid, acetylcholine, serotonin, and norepinephrine; modulate inflammatory responses through cytokines and immune cell activation; and regulate metabolic processes via short-chain fatty acids, bile acids, and other microbial metabolites. Collectively, the evidence reviewed herein does not suggest that the study of the gut microbiota has fundamentally altered the core pathophysiological understanding of MDD, which continues to center on monoaminergic dysregulation, HPA axis dysfunction, and neuroinflammation. Rather, it extends these established frameworks by identifying the gut microbiota as a convergent modulatory hub that may simultaneously influence neurotransmitter synthesis, immune homeostasis, and metabolic signaling. Thus, the microbiota–gut–brain axis offers a complementary mechanistic lens through which previously discrete pathophysiological processes can be viewed as functionally interconnected, pending further causal validation in human populations.

## Figures and Tables

**Table 1:** Gut Microbial Synthesis of Neuroactive Compounds and Their Effects on Depression.

Microbial Genus	Neuroactive Compound(s)	Mechanism of Action	Effect on Depression	Brain Region/Receptor	Reference
*Lactobacillus*	GABA, Acetylcholine	Activates vagus nerve, inhibits HPA axis	Reduces anxiety-like behavior	Hippocampus, Prefrontal cortex	[28,29,30]
*Bifidobacterium*	GABA, Acetylcholine	Enhances GABAergic inhibition	Alleviates depressive behavior	Hippocampal GABA receptors	[30,31]
*Escherichia*	5-HT, Dopamine, Norepinephrine	Activates 5-HT receptors, modulates HPA axis	Relieves depressive symptoms	Raphe nuclei, Prefrontal cortex	[32,33,34]
*Streptococcus*	Dopamine, Norepinephrine	Enhances noradrenergic signaling	Improves mood regulation	Locus coeruleus	[35]
*Enterococcus*	5-HT, Norepinephrine	Activates 5-HT1A receptors	Antidepressant-like effects	Hippocampus, Amygdala	[14]

Abbreviations: GABA, γ-aminobutyric acid; 5-HT, 5-hydroxytryptamine; HPA, hypothalamic–pituitary–adrenal; 5-HT1A, 5-hydroxytryptamine receptor 1A.

**Table 2:** Gut microbes influence host substances that affect depression.

Main Bacterium	Signal Molecule(s)	Host Product ↑	Host Product ↓	Reference
*Faecalibacterium prausnitzii*	Butyrate	BDNF, Tight-junction proteins	IL-6, TNF-α	[58]
*Bifidobacterium longum*	Acetate, GABA	Hippocampal GABA, IL-10	Corticosterone, IL-6	[59,60]
*Lactobacillus rhamnosus* GG	GABA, Indole-3-lactate	Plasma GABA, IFN-γ	Corticosterone	[61,62,63]
*Bacteroides thetaiotaomicron*	Zwitterionic polysaccharide	IL-10, Treg cells	Pro-inflammatory cytokines	[64]
*Roseburia intestinalis*	Butyrate	Colonic 5-HT, Tph1 expression	—	[58]
*Akkermansia muciniphila*	Propionate, AmEV vesicles	Tight-junction proteins	LPS translocation, Microglial activation	[65]
*Clostridium butyricum*	Butyrate, Secondary bile acids	BDNF, FXR activity	Neuro-inflammation	[66]
*Enterococcus faecalis*	Tyramine	Dopamine precursor pool	—	[67]

Abbreviations: GABA, γ-aminobutyric acid; BDNF, brain-derived neurotrophic factor; IL-6, interleukin-6; TNF-α, tumor necrosis factor-alpha; IL-10, interleukin-10; Treg, regulatory T; 5-HT, 5-hydroxytryptamine; Tph1, tryptophan hydroxylase 1; IFN-γ, interferon-gamma; AmEV, Akkermansia muciniphila extracellular vesicles; LPS, lipopolysaccharide; FXR, farnesoid X receptor. Note: ↑, increase/upregulation; ↓, decrease/downregulation.

## Data Availability

No new data were created or analyzed in this study. Data sharing is not applicable to this article.
